# Prey and predator density‐dependent interactions under different water volumes

**DOI:** 10.1002/ece3.7503

**Published:** 2021-03-30

**Authors:** Ross N. Cuthbert, Tatenda Dalu, Ryan J. Wasserman, Arnaud Sentis, Olaf L. F. Weyl, P. William Froneman, Amanda Callaghan, Jaimie T. A. Dick

**Affiliations:** ^1^ GEOMAR Helmholtz‐Zentrum für Ozeanforschung Kiel Kiel Germany; ^2^ Institute for Global Food Security, School of Biological Sciences Queen's University Belfast Belfast UK; ^3^ South African Institute for Aquatic Biodiversity Makhanda South Africa; ^4^ School of Biology and Environmental Sciences University of Mpumalanga Nelspruit South Africa; ^5^ Department of Zoology and Entomology Rhodes University Makhanda South Africa; ^6^ INRAE Aix Marseille University, UMR RECOVER Aix‐en‐Provence France; ^7^ DSI/NRF Research Chair in Inland Fisheries and Freshwater Ecology South African Institute for Aquatic Biodiversity Makhanda South Africa; ^8^ Ecology and Evolutionary Biology, School of Biological Sciences University of Reading Reading UK

**Keywords:** antagonism, consumer–resource, functional response, multiple predator effects, temporary pond, zooplankton

## Abstract

Predation is a critical ecological process that directly and indirectly mediates population stabilities, as well as ecosystem structure and function. The strength of interactions between predators and prey may be mediated by multiple density dependences concerning numbers of predators and prey. In temporary wetland ecosystems in particular, fluctuating water volumes may alter predation rates through differing search space and prey encounter rates. Using a functional response approach, we examined the influence of predator and prey densities on interaction strengths of the temporary pond specialist copepod *Lovenula raynerae* preying on cladoceran prey, *Daphnia pulex*, under contrasting water volumes. Further, using a population dynamic modeling approach, we quantified multiple predator effects across differences in prey density and water volume. Predators exhibited type II functional responses under both water volumes, with significant antagonistic multiple predator effects (i.e., antagonisms) exhibited overall. The strengths of antagonistic interactions were, however, enhanced under reduced water volumes and at intermediate prey densities. These findings indicate important biotic and abiotic contexts that mediate predator–prey dynamics, whereby multiple predator effects are contingent on both prey density and search area characteristics. In particular, reduced search areas (i.e., water volumes) under intermediate prey densities could enhance antagonisms by heightening predator–predator interference effects.

## Graphical abstract

Predator and prey densities are variable spatiotemporally across all habitat types globally. Our results indicate that predatory interaction strengths differ depending on densities of both predator and prey participants, with antagonistic multiple predator effects enhanced at intermediate prey densities. Future habitat changes resulting from anthropogenic activity that alter communities could have marked implications for trophic dynamics.

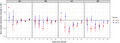

## 1 INTRODUCTION

Interspecific interactions mediate resource population stabilities, with implications for ecosystem structure and function (Brooks & Dodson, 1965; Paine, 1980; Wasserman & Froneman, 2013). Interaction strengths between consumers and resources, such as predators and prey, may be influenced by numerous biotic and abiotic environmental contexts that can challenge predictions of interaction strengths due to emergent effects (Cuthbert, Wasserman, et al., 2020; Sentis et al., 2019; Wasserman, Alexander, Dalu, et al., 2016), such as multiple predator effects (Barrios‐O'Neill et al., 2014; Sih et al., 1998). Predator effects can be additive (i.e., predator–predator independence), whereby consumption rates are predictable based on feeding rates of individuals (Cuthbert et al., 2020). In contrast, multiple predator effects can manifest as antagonistic (e.g., via predator–predator interference) or synergistic (e.g., via predator–predator cooperation) outcomes, which lessen and heighten prey risk, respectively (Sih et al., 1998; Vance‐Chalcraft & Soluk, 2005; Wasserman, Alexander, Dalu, et al., 2016). Evidence for the importance of trait‐mediated interactions such as multiple predator effects in influencing population dynamics is increasing (Anton et al., 2020; Gabowski, 2004; Schmitz et al., 2004; Trussell et al., 2004). In particular, trait‐mediated effects in aquatic ecosystems have been found to be pervasive due to the presence of waterborne cues that illicit responses over entire populations (Alexander et al., 2013; Peacor & Werner, 2001; Trussell et al., 2004, 2008).

Multiple predator effects have been classically quantified by measuring predatory interactions toward one or just a few prey densities, using experimental designs that considered interspecific but not intraspecific consumers (Soluk, 1993; but see Skalski & Gilliam, 2001; Rall et al., 2008). Accordingly, there has been a relative lack of examination of potential conspecific multiple predator effects, despite the importance of intraspecific interactions in certain habitat types (Cuthbert, Dalu, Wasserman, Weyl, et al., 2020). Indeed, studies have found intraspecific interference to be common across many study systems, and these tend to be intermediate in magnitude overall (−0.6 to −0.7 m), despite high variability among studies (DeLong & Vasseur, 2011).

In addition to predator density effects, predator–prey interactions are known to be inherently prey density‐dependent (Dick et al., 2014; Holling, 1959). Differences in prey density dependence forms can be driven by prey refuge effects that can arise at low‐density prey populations (Alexander et al., 2012). For example, predators can target prey even when relatively rare in environments, promoting their extirpation. Conversely, predators can avoid rare prey and instead predate abundant groups, helping to stabilize populations and minimize extirpations through frequency‐dependent predation (Murdoch, 1969; Murdoch et al., 1975). Classically, the “functional response” has been used to quantify rates of resource consumption as a function of resource density (Holling, 1959). Functional responses are characterized into three forms: types I (i.e., linear), II (i.e., hyperbolic), and III (i.e., sigmoid) (Hassell, 1978). Type I functional responses are mechanistically restricted to filter feeders owing to an absence of resource search‐related constraints (Jeschke et al., 2004). Type II functional responses can be stabilizing compared with type I due to consumption limitations at high‐resource densities. Conversely, type II functional responses can be destabilizing compared with type III owing to high consumption rates at low prey densities, while consumption rates are lower in type III functional responses at low prey densities (Dick et al., 2014; Hassell, 1978). The type III response also prevents prey from reaching high densities where population fluctuations are more likely to occur. Furthermore, recent advances have applied functional responses for the discernment of multiple predator effects across a range of resource densities, with several methods applied (e.g., multiplicative risk model: Wasserman, Alexander, Dalu, et al., 2016; population dynamic model: Sentis & Boukal, 2018). Indeed, functional responses are known to be both predator and prey density‐dependent (Abrams & Ginzburg, 2000; Coblentz & DeLong, 2020). Fundamentally, examinations of multiple predator effects using functional responses enable quantification of effects of both predator and prey density dependences simultaneously, as well as potential interaction effects among these factors. Previous works concerning multiple predator effects have shown significant dependence on prey availability, as well as other factors such as climatic warming and body size (Dalal et al., 2020; Sentis et al., 2016).

Functional responses have been recently applied to quantify interaction strengths within temporary pond ecosystems in arid environments (Buxton et al., 2020; Cuthbert et al., 2018; Wasserman, Alexander, Barrios‐O'Neill, et al., 2016). Trophic structuring in these systems is atypical and can be determined by phenologies of dormant eggs, which hatch when ponds fill with water and the hydroperiod begins (Wasserman, Alexander, Barrios‐O'Neill, et al., 2016). Communities in these systems are highly biodiverse (Bird et al., 2019) and increasingly threatened by anthropogenic global change (Dalu, Wasserman, & Dalu, 2017). In austral environments, temporary ponds are poorly studied despite their suitability as model systems in ecology for testing theories (De Meester et al., 2005). Predation pressure in these systems is transient throughout the hydroperiod, with higher predators such as hexapods arriving late in the hydroperiod via aerial dispersal (Wasserman et al., 2018). Accordingly, for much of the early hydroperiod stages, or throughout during short wet phases, predator assemblages are dominated by abundant zooplankton species owing to rapid mass hatching events following inundation. Interspecific interactions in these systems among conspecific predatory zooplankton can thus be marked, as such consumers are under temporal pressures to utilize resources to enable rapid development and maturation to reproduce prior to the hydroperiod ending (i.e., pond drying) (Dalu, Wasserman, Vink, et al., 2017). However, despite high abundances of zooplankton in these systems and spatiotemporally changeable population dynamics, few works have examined the potential for conspecific multiple predator effects as predator and prey densities simultaneously shift (Cuthbert, Dalu, Wasserman, Weyl, et al., 2020).

Naturally, water volumes are extremely variable in temporary pond ecosystems owing to wetting and drying, which can mediate interaction strengths owing to differences in search areas and encounter rates with prey or other predators (Uiterwaal et al., 2018; Uiterwaal & DeLong, 2018). Moreover, anthropogenic processes such as water extraction could also alter the volume of ponds, particularly as water shortages become more severe (Dalu, Wasserman, & Dalu, 2017). Reductions in search area might heighten encounter rates with prey by improving search efficiencies, and yet could also drive greater interference or mutualistic effects between interacting predators (i.e., multiple predator effects). The present study thus examined the influence of water volume on emergent multiple predator effects between conspecifics of the calanoid copepod *Lovenula raynerae* Suárez‐Morales et al. (2015), preying on representative cladoceran prey. This copepod species has been identified as an important and abundant predator in temporary pond ecosystems, where cladocerans such as daphniids coexist with predatory copepods due to simultaneous resting egg hatching (Cuthbert et al., 2018; Wasserman, Alexander, Barrios‐O'Neill, et al., 2016; Wasserman et al., 2018). We systematically altered predator abundances using an additive experimental design, while also varying water volumes and prey densities factorially. We then used a comparative functional response approach, combined with a population dynamic model, to predict intraspecific multiple predator effects as population densities shift.

## 2 MATERIALS AND METHODS

Adult male *L. raynerae* (4.5–5.0 mm) (Suárez‐Morales et al., 2015) and mature *Daphnia pulex* complex (2.2–2.6 mm) were collected from a temporary pond close to Makhanda in the Eastern Cape Province of South Africa (33°15′04.1″S 26°26′17.0″E) by hauling a zooplankton net through the water column. Zooplankton were transported in source water to the Department of Zoology and Entomology, Rhodes University, separated, and housed in a controlled environment room (21 ± 1°C; 12:12 light:dark phase) in 5‐L tanks containing filtered (20 μm sieved) source water. Copepods were unfed for 24 hr before use to standardize levels of hunger.

Feeding rates of *L. raynerae* were quantified toward daphniid prey under two levels of water volume and four predator densities. Prey were introduced at one of six densities (2, 4, 8, 16, 32, and 64) into 100‐ml experimental arenas (5.6 cm diameter) containing 40 or 80 ml of filtered source water. After settling for 2 hr, one of four predator densities (1, 2, 3, or 4 ind.) was introduced into experimental arenas, with predators allowed to feed for 18 hr. Five replicates were undertaken per experimental group (i.e., 240 units overall; 6 prey densities × 2 volumes × 4 predator densities × 5 replicates), and the design was fully randomized to avoid positional effects. Five replicates of predator‐free controls were also run at each prey density and water volume to quantify background prey mortality rates. After the allocated feeding period, predators were removed and remaining live prey counted to quantify numbers killed. Predators were not used in more than one experimental trial to standardize prior predator experience and avoid the potentially confounding factor of predator identity.

Differences in the proportions of prey eaten were analyzed using quasi‐binomial generalized linear models (owing to residual overdispersion) as a function of water volume, predator density and prey density, and their interactions. Nonsignificant terms were removed from the model such that the final model contained only significant predictors (Crawley, 2007). Post hoc pairwise tests were computed using Tukey's comparisons.

Binomial generalized linear models were additionally used to categorize functional response types for each depth treatment at the single‐predator density (i.e., predator density = 1 predator; Juliano, 2001; Pritchard et al., 2017). A type II functional response was indicated through the presence of a significantly negative linear coefficient in response to increasing prey density. Given that prey were not replaced following consumption over the course of the experiment, Rogers' random predator equation was used to model functional responses at the single‐predator densities (Rogers, 1972):

(1) $$N_{e}=N_{0}\left(1‐\exp\left(a\left(N_{e}h‐T\right)\right)\right)$$

where *N_e_* is the number of prey eaten, *N*_0_ is the initial density of prey, *a* is the attack constant, *h* is the handling time, and *T* is the total experimental period. The Lambert W function was used to fit the model to the data (Bolker, 2008; Pritchard et al., 2017). The random predator equation is robust to prey depletion in parameter estimation (Cuthbert, Wasserman, et al., 2020). Total prey depletion occurred across all replicates in the following two treatments: at 1 predator, 40 ml volume, 2 prey; and at 3 predators, 80 ml volume, 2 prey. Attack rates and handling times were compared using the differences (delta) method outlined in Juliano (2001), pairwise between each water volume.

**FIGURE 1:**
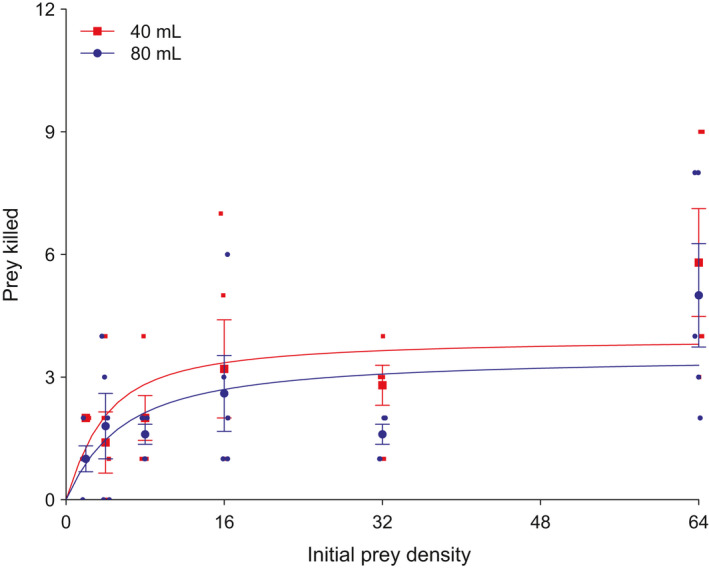
Functional responses of *Lovenula raynerae* under single‐predator densities between water volumes. Points are means ± 1 standard error, and smaller points are raw data. Note that the curves were modeled using data from all predators within a given treatment. The solid lines show the functional responses modeled from the random predator equation

We quantified interaction strength (IS) as the proportion of prey killed at each predator density, water volume, and prey density by dividing the number of prey consumed by the initial prey density (Veselý et al., 2019):

(2) $$\text{IS}\left(P,\;Z\right)=\frac{N_{P}‐N_{P,Z}}{N_{P}}$$

where *N_P_* and *N_P,Z_* are the numbers of live prey at the beginning and end of the experiment, respectively. The proportion of prey killed (IS) includes both trophic interactions (i.e., feeding on prey) and nontrophic interactions that can enhance (e.g., facilitations among predators) or reduce (e.g., interference among predators) trophic interactions. To disentangle trophic (IS_T_) and nontrophic (IS_NT_) interactions, we next used a population dynamic approach that quantifies IS_NT_ as the difference between the observed IS and the predicted IS from single‐predator functional responses (Sentis et al., 2017). Accordingly, we used our attack rate and handling time estimates from single‐predator functional responses to predict multiple predator feeding rates, which were then compared with observed multiple predator feeding rates. This was done separately using functional response parameters for each water volume using the corresponding single‐predator functional response parameters. Estimations of IS_T_ were calculated following McCoy et al. (2012) and Sentis and Boukal (2018):

(3) $$\frac{\mathrm{dN}}{\mathrm{dt}}=‐\frac{\mathrm{aN}}{1+ahN}P$$

where *N* is the prey population density, *P* is the predator population density, and *a* and *h* are the attack rate and handling time obtained from the single‐predator functional response estimates. This model assumes no emergent multiple predator effects, and its predictions can be compared with multiple predator feeding trials to assess the sign and strength of multiple predator effects. To generate predictions of expected prey survival in the multipredator experiments, initial values of *N* and *P* are set at the experimental initial prey and predator densities corresponding to the experimental treatment. For each predator density, water volume, and prey density, Equation 3 was then integrated over the full experimental time to obtain expected numbers of surviving prey. To estimate the variance around the predictions, we used a global sensitivity analysis that uses the 95% confidence intervals of each functional response parameter estimate and their variance–covariance matrix (covariance is assumed to be zero when unknown) to generate 100 random parameter sets using a Latin hypercube sampling algorithm (Soetaert & Petzoldt, 2010). For each parameter set (*n* = 100), Equation 3 is then integrated over time and expected prey survival calculated using the “sensRange” function in the R package “FME” (Soetaert & Petzoldt, 2010). In turn, these numbers of surviving prey predicted were used reciprocally to determine the numbers of prey killed at each multiple predator density, water volume and prey density, and thus the proportion of prey killed per treatment for comparison with IS (Equation 2). Multiple predator effects, including interference effects (i.e., IS_NT_), were then calculated by subtracting the mean IS_T_ (i.e., predictions) from IS (i.e., observations). We note that variance in our prediction of IS_NT_ at predator densities of 1 was due to differences (i.e., error) between the modeled functional responses and underlying consumption data (see Figure 1). Furthermore, the IS_T_ predictions were made on a continuous scale, whereas IS data (proportion killed) are based on discrete counts of prey killed. This may lead to some divergence between predictions and observations, especially at low prey densities where continuous and discrete scales are more likely to differ and thus be more sensitive to less accurate model fitting. Positive and negative values of IS_NT_ correspond to prey risk enhancement and reduction, respectively. Multiple predator effects were analyzed using linear models. Homogeneity of variances and residual normality were examined. A backward step deletion process was employed to obtain the most parsimonious model through removal of nonsignificant terms and interactions, as before (Crawley, 2007). We compared models with the prey density covariate included as linear and quadratic terms via AICc, to select the model that minimized information loss. Tukey's tests were used post hoc for comparison of levels within significant effects.

## 3 RESULTS

Most control prey survived across predator‐free treatments (96% survival, ±8% *SD*), and thus, no adjustments were made to experimental mortality rates. Predation rates differed significantly according to predator density and prey density (Table 1); there was no significant effect of water depth on total consumption rates, nor any significant two‐ or three‐way interaction terms. With the exception of 2 versus 3 predators (*p* > 0.05), total feeding rates always significantly increased with increasing predator densities (all *p* < 0.05). Predation rates fell significantly with increasing prey density (Table 1).

**TABLE 1:** Generalized linear model results considering feeding rates by multiple *Lovenula raynerae* as a function of predator density, water volume, and prey density. Significant predictors are emboldened

Predictor	*F*‐value (*df*)	*p*‐value
**Predator density**	**18.79 (3, 236)**	**<0.001**
Volume	0.02 (1, 235)	0.90
**Prey density**	**247.53 (1, 234)**	**<0.001**
Predator density × volume	0.50 (3, 231)	0.68
Predator density × prey density	1.40 (3, 228)	0.24
Volume × prey density	1.34 (1, 227)	0.24
Predator density × volume ×prey density	0.03 (3, 224)	0.99

The proportion of prey consumed was negatively related to initial prey density, and thus, functional responses were categorized as type II at the single‐predator density for both water volume treatments (Table 2; Figure 1). Attack rates tended to be higher, and handling times shorter, in the low‐ compared with high‐volume treatments. However, neither attack rates nor handling times differed significantly pairwise according to volume treatments at each predator density (attack rate, *z* = 0.73, *p* > 0.05; handling time, *z* = 0.41, *p* > 0.05).

**TABLE 2:** Functional response linear coefficients (and *p*‐values), attack rates, and handling times across predator density and water volume treatments alongside standard errors (*SE*)

Predator density	Volume (ml)	Linear coefficient, *p*‐value	Attack rate, *SE*	Handling time, *SE*
1	40	−0.03, <0.001	1.52, 0.93	0.25, 0.05
1	80	−0.03, <0.001	0.79, 0.38	0.28, 0.06

**FIGURE 2:**
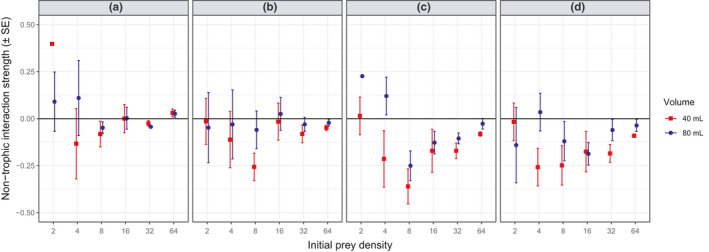
Predicted nontrophic interaction strength (i.e., multiple predator effects) of (a) one, (b) two, (c) three, and (d) four *Lovenula raynerae* predators between water volumes and across prey densities. Negative values are indicative of prey risk reduction, while positive values indicate prey risk enhancement

Multiple predator effects were predominantly antagonistic (i.e., negative IS_NT_) among conspecific *L. raynerae* overall (Figure 2), indicating prey risk reductions in multiple predator groupings. Multiple predator effects differed significantly among predator densities (*F*_3,233_ = 5.16, *p* < 0.01), whereby predator densities of 3 and 4 were significantly more antagonistic than densities of 1 overall (both *p* < 0.05). The strength of negative multiple predator effects among *L. raynerae* was also significantly greater under reduced water volumes overall (*F*_1, 233_ = 5.32, *p* < 0.05), whereby greater single‐predator attack rates at reduced volumes (Table 2) caused higher predictions, and thus observed/predicted divergence, at low prey densities. Inclusion of prey density as a quadratic term improved the model as compared to a linear term alone (ΔAICc = 3.88; linear, *t* = 0.12, *p* > 0.05; quadratic, *t* = 2.43, *p* < 0.05), indicating that antagonistic multiple predator effects scaled unimodally at intermediate prey densities. The strength of antagonisms tended to be greatest around prey densities of 8 (Figure 2). There were no significant interaction terms (all *p* > 0.05).

## 4 DISCUSSION

The present study found density and water volume‐dependent multiple predator effects to mediate predator–prey interactions between temporary pond specialists. While feeding rates were found to increase overall under increasing predator densities, negative multiple predator effects pointed to increasing interference effects among conspecific predators, and thus reduced per capita consumption. These negative effects were, however, most prevalent under reduced water volumes, indicating that smaller search areas could exacerbate antagonistic interactions that alleviate prey risk. In ephemeral ponds that are subject to changeable water volumes, this could result in changeable multiple predator effects as ponds dry. Furthermore, while feeding rates at low prey densities were not suppressed (i.e., type II functional responses), the magnitude of multiple predator effects peaked at intermediate prey densities, suggesting that the availability of prey in environments can further influence predator–predator interactions. This finding corroborates previous studies that have found multiple predator interactions to be prey density‐dependent (Sentis et al., 2016). Centrally, as predators seldom occur in individually in ecosystems, understanding multiple predator effects is critical for predicting interaction strengths holistically (Cuthbert, Dalu, Wasserman, Weyl, et al., 2020; Griffin et al., 2013; Sih et al., 1998; Wasserman, Alexander, Dalu, et al., 2016). For example, these quantifications could be used to better understand the implications of predator extirpation for trophic dynamics within ecosystems, or the effects of hydrological alterations following environmental change in aquatic systems. In tropical waters, increasing droughts may reduce water volumes in temporary ponds due to higher water demands and changing precipitation patterns, or could result in their increase via deepening through land modifications for water storage purposes (Dalu, Wasserman, & Dalu, 2017).

Studies concerning multiple predator effects have reported a lack of generality among consumer–resource systems, with examples of antagonistic, additive, and synergistic outcomes in recent decades (Barrios‐O'Neill et al., 2014; Losey & Denno, 1998; Sih et al., 1998; Soluk, 1993; Vance‐Chalcraft & Soluk, 2005; Wasserman, Alexander, Dalu, et al., 2016). The lack of consistency suggests that multiple predator effects are system‐specific and may be mediated by context‐dependent factors such as taxonomic grouping, population demographics, and habitat characteristics. However, the current sparsity of studies into multiple predator effects overall negates broader generalizations within or between such factors, particularly at the conspecific level. Recent works into another temporary pond specialist copepod, *Paradiaptomus lamellatus* Sars, 1985, has similarly found antagonistic interactions in calanoid copepods (Cuthbert, Dalu, Wasserman, Monaco, et al., 2020). This species is known to coexist with the focal species in the present study, *L. raynerae*, although it is less voracious (Wasserman, Alexander, Barrios‐O'Neill, et al., 2016). Interspecific multiple predator effects among temporary pond copepods have, however, previously been found to combine additively (Cuthbert, Callaghan, et al., 2020). Moreover, Buxton et al. (2020) found multiple predator effects of a congeneric copepod to combine additively, if not synergistically, with higher‐order predatory hexapods from temporary ponds. Accordingly, a variety of multiple predator effects have been reported in austral temporary pond ecosystems, with implications for prey population stability and “boom‐bust” population dynamics (Wasserman et al., 2018). Despite these recent advances, the influence of predator densities beyond pairs, as well as effects in combination with water depth variations, had yet to be considered prior to this study. Nonetheless, the present study found the multiple predator effect to be strongest at predator densities beyond two, with relatively little further reduction in effects between densities of three and four. The increasing intensities of nontrophic interactions at reduced water volumes found in the present study likely emanate from greater predator–predator encounter rates, which interfere with predatory efficiencies, yet the mechanisms underlying this require further elucidation empirically at a broader range of volumes.

Prey density implications for multiple predator effects have been poorly studied until recently. Previous studies have identified intraguild predation (i.e., interactions among predators) to lessen under increasing prey densities (Lucas & Rosenheim, 2011; Sentis et al., 2013). In agreement with the finding of this study where multiple predator effects were highest at intermediate prey densities, Sentis et al. (2016) found unimodal scaling of multiple predator effects. This patterning might arise from the interplays between predation rates and predator satiation. At low prey densities, most if not all prey are consumed, which masks any potential interference effects in nonreplacement experimental feeding designs. Conversely, at high prey densities, not all prey are consumed, and thus high predator satiation minimizes multiple predator interference with one another. The comparative functional response approach thus lends itself to studies of multiple predator effects, as they are able to capture effects across a range of resource densities, which were not considered in many previous studies (Griffen, 2006; Soluk, 1993; Tylianakis & Romo, 2010; Vance‐Chalcraft & Soluk, 2005). This thus enhances the resolution of results across different population abundance scenarios.

In the present study, owing to the lack of interaction effects, this unimodal patterning where antagonisms peaked at intermediate prey levels was consistent irrespective of predator density or water volume. However, the latter also exacerbated multiple predator effects where water volumes were reduced. In the case of water volume, reductions in search area associated with reduced water levels likely increase encounter rates among predators, and particularly for actively pelagic consumers, such as *L. raynerae* in the present study. Copepods utilize hydromechanical cues, detected by mechanoreceptors on the antennules, for the detection of predators and prey (Hwang & Strickler, 2001). While considered the most important cue factor for copepod predator–prey interactions, and with evidence suggesting that copepods can differentiate between signals from predators and prey (Hwang & Strickler, 2001), conspecific hydromechanical cue recognition dynamics are unknown. Reduced proximities between conspecifics in association with increased individual numbers, or decreased water volumes, may result in increased hydromechanical signal reception. If these signals are indistinguishable from predator cues, crowding may result in increases in anti‐predator responses, with implications for foraging time and efficacy at reduced water volumes. Alternatively, antagonistic effects might merely reflect an increase in predator–predator encounters and thus interference. These effects may be less pronounced for benthic ambush predators, which might exhibit lower predator–predator encounters. Indeed, Sentis et al. (2016) found multiple predator effects to differ markedly depending on the predator assemblage characteristics. In the context of functional responses, previous works have also found arena size to significantly alter the scaling of parameters (attack rate and handling time) (Uiterwaal et al., 2018; Uiterwaal & DeLong, 2018); thus, in a similar vein, we suggest that consideration for search area is critical in studies into multiple predator effects in aquatic systems.

Austral temporary ponds lack examination as they remain inconspicuous during dry periods. This is despite them being threatened by a number of anthropogenically mediated processes, including climate and land use changes (Dalu, Wasserman, & Dalu, 2017). In particular, increased droughts, pollutants and hydrological alternations *via* water extraction threaten these systems and the biodiversity they support (Mabidi et al., 2018). Many temporary ponds are not mapped let alone studied, and understandings of trophic dynamics in these systems are lacking. Predatory copepods, such as *L. raynerae*, are often highly abundant in temporary ponds in the study region (Wasserman et al., 2018). These copepods hatch early in the hydroperiod from drought‐resistant eggs, and thus have high potential to engage in predator–predator interactions with conspecifics. The focal prey in this study, *D. pulex,* also hatch from dormant eggs and cooccur with copepods, and thus, our predator–prey system is representative of multiple community composition scenarios considering predator and prey densities. This is particularly the case given that food webs in these systems can be highly simplified, particularly during the early stages of hydroperiod where recruitment is largely restricted to internal hatching events. Overall, the results from the present study advance our understandings of predator and prey density dependences of inter‐ and intraspecific interactions in these ecosystems, and also indicate how differences in water depth alter the nature of trophic dynamics. Reductions in water depth through the hydroperiod likely exacerbate predator–predator interactions, and thus may have implications for prey population stability. Future studies are required to examine other trait‐mediated effects in these systems, as well as for considering more complex assemblages of trophic groups and additional environmental variables. Empirical work that considers a broader range of water volumes is also required to further our understandings of multiple predator effects in aquatic ecosystems.

## CONFLICT OF INTEREST

There are no conflicts of interest relating to this study.

## AUTHOR CONTRIBUTION

**Ross N. Cuthbert:** Conceptualization (lead); Data curation (lead); Formal analysis (lead); Investigation (lead); Methodology (lead); Visualization (lead); Writing‐original draft (lead). **Tatenda Dalu:** Conceptualization (equal); Writing‐review & editing (equal). **Ryan J. Wasserman:** Conceptualization (equal); Writing‐review & editing (equal). **Arnaud Sentis:** Formal analysis (equal); Software (equal); Writing‐review & editing (equal). **Olaf L. F. Weyl:** Project administration (equal); Resources (equal); Writing‐review & editing (equal). **P. William Froneman:** Resources (equal); Writing‐review & editing (equal). **Amanda Callaghan:** Supervision (equal); Writing‐review & editing (equal). **Jaimie T. A. Dick:** Supervision (equal); Writing‐review & editing (equal).

## Supporting information

Data S1

## Data Availability

Underlying data are available on the Dryad digital repository (https://doi.org/10.5061/dryad.qz612jmf5).
